# Blood group antigens SLeX, SLeA, and LeY as prognostic markers in endometrial cancer

**DOI:** 10.1007/s00432-022-04098-8

**Published:** 2022-06-21

**Authors:** Thomas Kolben, Lena Müller, Sarah Meister, Lucia Keilmann, Christina Buschmann, Fabian Trillsch, Alexander Burges, Bastian Czogalla, Sophie Mitter, Elisa Schmoeckel, Stefanie Corradini, Sven Mahner, Udo Jeschke, Mirjana Kessler, Susanne Beyer

**Affiliations:** 1 Department of Obstetrics and Gynecology, University Hospital, LMU Munich, Marchioninistr. 15, 81377 Munich, Germany; 2 Institute of Pathology, University Hospital, LMU Munich, Marchioninistr. 15, 81377 Munich, Germany; 3 Department of Radiation-Oncology, University Hospital, LMU Munich, Munich, Germany; 4 Department of Obstetrics and Gynecology, University Hospital, Universitätsklinikum Augsburg, Stenglinstr. 2, 86156 Augsburg, Germany

**Keywords:** Sialyl Lewis X, Sialyl Lewis A, Lewis Y, Endometrial cancer, Survival

## Abstract

**Purpose:**

Endometrial cancer (EC) is the most common gynecological cancer worldwide. Treatment has been improved in recent years, but, in advanced stages, therapeutical options are still limited. It has been reported that the expression of the blood group antigens Sialyl Lewis X (SLeX), Sialyl Lewis A (SLeA) and Lewis Y (LeY) is associated with prognosis in several tumors. Large studies on endometrial and cervical cancer are still pending.

**Methods:**

Specimens of 234 patients with EC were immunohistochemically stained with antibodies for SLeX, SLeA and LeY. Expression was correlated to histopathological variables.

**Results:**

High expression of SLeX was correlated to low pT-stage (*p* = 0.013), low grade (*p* < 0.001), low FIGO-stage (*p* = 0.006) and better overall survival rates (OS; *p* = 0.023). High expression of SLeA was associated with low pT-stage (*p* = 0.013), low grade (*p* = 0.001) and better progression-free survival (PFS; *p* = 0.043). LeY staining was correlated to pN + (*p* = 0.038), low grade (*p* = 0.005) and poorer PFS (*p* = 0.022).

**Conclusion:**

This is the first study examining the expression of SLeX, SLeA and LeY in EC, which can serve as additional future prognostic markers. Further studies are necessary to understand the underlying mechanisms. The study was approved by the local ethics committee of the Ludwig-Maximilians University Munich (reference number 19-249).

**Supplementary Information:**

The online version contains supplementary material available at 10.1007/s00432-022-04098-8.

## Introduction

Endometrial cancer (EC) is still an illness with high importance for global health initiatives: it is the sixth most common cancer among women worldwide (Bray et al. 2018). The *International Agency for Research and Cancer* estimated an incidence of 382.069 cases and a mortality of almost 90.000 worldwide in 2018 (Bray et al. 2018), and an increasing incidence is prognosticated (Society 2014). A relatively high exposition to estrogen is the main risk-factor for EC, including early menarche, therapy with tamoxifen, nulliparity, diabetes or obesity (Braun et al. 2016). Due to these risk factors, EC was originally distinguished in estrogen-dependent (Typ I) and estrogen-independent (Type II) types (Bokhman 1983). Due to improved molecular examinations today, EC is now classified according to the ProMisE-algorithm, containing MMR-deficiency, POLE mutation, p53 wildtype and p53 aberrancy (Kommoss et al. 2018; Kandoth et al. 2013). Information of this classification has recently been acknowledged in the most recent treatment recommendations addressing the different treatment modalities (surgery, radiotherapy, chemotherapy) (Concin et al. 2021). Nevertheless, about 15% of all EC patients experience a recurrence. In these situations, new therapies with checkpoint inhibitors like Pembrolizumab have been licensed (Arora et al. 2020). But for many patients sufficient therapeutic options are still missing, so that new markers are needed (Legge et al. 2020). For this reason, oncological researchers focused on several blood group antigens, whereas data in EC are still missing.

Blood group antigens, like AB0 or Lewis antigens do not only characterize red blood cells. They are also involved in signaling pathways, cell adhesion and recognition as well as signal transduction (Phillips et al. 1990; Crucho et al. 2015). By these functions, they play an important role in tumorigenesis (Lin et al. 2009). Lewis antigens are carbohydrate antigens on the cell surface and they are markers for cell differentiation in fetal cells (Liu et al. 2018; Ugorski and Laskowska 2002). In adult humans, they are present in several tissues and they also can be tumor-associated (Liu et al. 2018; Ugorski and Laskowska 2002). Well-known representatives are Sialyl Lewis X (SLeX), Sialyl Lewis A (SLeA) and Lewis Y (LeY). Overexpression of the Lewis antigens SLeX, SLeA and LeY have been documented in several cancer cells (Ugorski and Laskowska 2002; Iwanari et al. 1990; Madjd et al. 2005). LeY is fucosylated (Fucα1-2Galβ1-4[Fucα1-3]GlcNAc) (Liu et al. 2018), while SLeX (Siaα2,3Galβ1,4[Fucα1,3]GlcNAc) and SLeA (Siaα2,3Galβ1,3 [Fucα1,4]GlcNAc) are two glycoconjugated isomers (Trinchera et al. 2017). Glycoconjugates are essential for inter-cell communication and for the interaction with the cell environment. As surface marker of leukocytes, they are also essential for the immune system, a loss of SLeX can lead to severe immune defects (DeLisser et al. 1999). Via the described functions, the Lewis antigens are essential for cell signaling (Liu et al. 2019), including immune system, cancerogenesis, cancer invasion and metastasis (Trinchera et al. 2017). In cancer cells, the structure of carbohydrates changes, including glycosylation of SLeX or SLeA, e.g., (Dall’Olio 1996; Roseman 2001). Although glycosylation seems to be important in carcinogenesis, the process of glycosyltransferase gene regulations and its relation to cancer is still unknown, whereas epigenetic processes seem to be involved (Lauc et al. 2014). An elevated expression of Lewis antigens was detected in EC (compared to healthy endometrial tissue), but no analysis regarding histological parameters and survival data has been performed (Tsukazaki et al. 1991), so far. It seems clear, however, that Lewis antigens play an important role in carcinogenesis and the immune system, which makes them interesting for EC, an immunogenic cancer.

Therefore, the aim of this study was to examine SLeX, SLeA and LeY in endometrial cancer and to analyze their correlation to histopathological parameters and survival data.

## Materials and methods

### Materials

For our study, we obtained 234 endometrial cancer samples from patients who were treated by surgery in the Department of Obstetrics and Gynecology of the Ludwig-Maximilians-University Munich between 1990 and 2001 due to available survival data. The material was embedded in paraffin and prepared as tissue-micro-arrays (TMA) by the LMU Pathology Institute. An overview of the distribution of the clinic pathological parameters in this study group is given in Table 1.

**Table 1:** Distribution of histopathological parameters

Item	No./total no	**%**
Age at diagnosis		
≤ 65	113/234	48.3
> 65	121/234	51.7
Tumor size, pT		
pT1	181/234	77.4
pT2	17/234	7.3
pT3	31/234	13.2
pT4	4/234	1.7
No information	1/234	0.4
FIGO		
I	172/234	73.5
II/III/IV	61/234	26.1
No information	1/234	0.4
Grading		
I	128/234	54.7
II	77/234	32.9
III	28/234	12
No information	1/234	0.4
Number of positive lymph nodes, pN		
pN0	147/234	62.8
pN1	22/234	9.4
pNx	65/234	27.8
Metastasis, pM		
pM0	111/234	47.8
pM1	6/234	2.6
No information	117/234	49.6
Histology		
Endometrioid	225/234	96.2
Clear cell	8/234	3.4
Mucinous	1/234	0.4
Therapy		
Surgery	134/234	57.3
Radiotherapy	1/234	0.4
Surgery + radiotherapy	89/234	38
Surgery + chemotherapy	2/234	0.9
Surgery + hormonal therapy	5/234	2.1
Surgery + radiotherapy + chemotherapy	3/234	1.3
Survival		
Died	97/234	41.5
Censured	137/234	58.5
Progression		
At least one	49/234	20.9
No information	185/234	79.1

### Ethics approval

The data were completely anonymized and identifying attributes were not accessible for the authors during experiments and analysis.

The principles of the Declaration of Helsinki with its amendment of Seoul 2008 were taken into account during the planning und conducting process. The study was approved by the local ethics committee of the Ludwig-Maximilians University Munich (reference number 19-249).

### Immunohistochemistry

For immunohistochemical staining, the TMAs (obtaining 3 tissue spots for each patient) were first deparaffinized in Roticlear and afterwards washed in 100% Ethanol. To block endogenous peroxidase, the samples were left for 20 min in a 1% Methanol/H2O2 solution. Subsequently, the slides were first dehydrated by rinsing in a descending ethanol series and later left for 5 min in a trisodium citrate buffer solution (pH = 6) in a pressure cooker to demask the antigens. Afterwards, the samples were washed in distilled water and PBS-buffer. These steps were the same for the staining of all four antibodies.

To avoid unspecific hydrophobic binding between immunoglobulins and tissue components, we applied a blocking solution to saturate electrostatic charges. An overview of the used chemicals is given in Tables 2 and 3. Afterwards, the slides were incubated with the primary antibodies.

**Table 2:** Overview of antibody and chemicals used in the staining process

Sialyl Lewis X	Sialyl Lewis A	Lewis Y
Blocking solution^a^: 20 min	Blocking solution^b^: 5 min	Blocking solution^c^: 3 min
Primary antibody^d^	Primary antibody^d^	Primary antibody^d^
1:200 in PBS^e^	1:80 in PBS^e^	1:50 in PBS^e^
Incubation 16 h, 4 °C	Incubation 60 min, 23 °C	Incubation 16 h, 4 °C
Secondary antibody^f^	PostBloc^a^k: 20 min	Secondary antibody^f^
1:50 in DAKO diluent^g^		1:200 in DAKO diluent^g^
Incubation 30 min, 23 °C		Incubation 30 min
ABC-complex^h^: 30 min	HRP Polymer^a^: 30 min	ABC-complex^h^: 30 min
Chromogen: DAB^i^, 47 s	Chromogen: DAB^i^, 43 s	Chromogen: DAB^i^, 4 min

^a^Vectastain Elite ABC Kit, diluted NORMAL serum

^b^ZytoChem Plus HRP Polymer Kit (Mouse/Rabbit) 3 × 100, Cat.No. POLHRP-100

^c^Universal Blocking Reagent (10X), REF HK085-5KE

^d^Information in Table 3

^e^Dulbecco’s phosphate-buffered saline

^f^Biotinylated Goat-anti-Mouse IgM, Linaris, Nr. ZMB2020

^g^DAKO Antibody Diluent with Background Reducing components, REF S3022

^h^Vectastain Elite ABC Kit, REAGENT A (Avidin, ABC Elite) 30,005, REAGENT B (Biotinylated HRP, ABC Elite) 30,006

^i^Liquid DAB + Substrate Chromogen System, REF K3468

**Table 3:** Primary antibodies

Antigen	Company	Antibody	Host	Synonyme	Catalog ID
Sialyl Lewis X	BD Pharmingen	Monoclonal IgM	Mouse	CD15s	551,344
Sialyl Lewis A	SIGMA-ALDRICH	Monoclonal IgM	Mouse	CA19.9	SAB4700773
Lewis Y	LSBio	Monoclonal IgM	Mouse	CD174	LS-C311942

After incubation and rinsing in PBS, staining was increased by two different methods. For the staining of SLeX and LeY, we used the ABC-method (avidin–biotin-method) whereas the SLeA staining was performed using amplifying PostBlock solution and applying HRP-Polymer.

The enzymatic color reaction was performed using DAB-Chromogen and followed by counterstaining with hemalun. Slides were dehydrated in a rising ethanol series and covered using RotiMount.

During the staining process, some samples pelt off, so the number of analyzed samples varied between 216 and 234 depending on the used antibody.

We evaluated the expression of SLeX, SLeA and LeY using the well-established semi-quantitative immunoreactive score (IRS). Therefore, intensity was rated between 0 and 3 (0 = no staining, 1 = low intensity, 2 = moderate intensity, 3 = high intensity). The percentage of the stained tumor cells was subdivided and rated as follows: 0 = 0%, 1 = 1–10%, 2 = 11–50%; 3 = 51–80%; 4 = 81–100%). The values for intensity and stained percentage were multiplied to obtain the IRS. This was done for each spot on the TMA individually. We used the mean of all three scores to assess the final IRS for each patient.

**Fig. 1:**
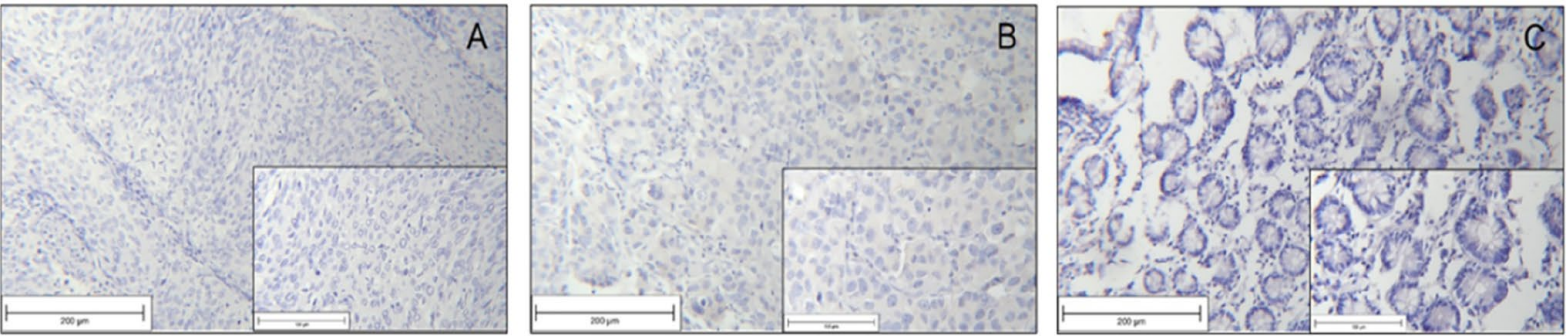
Negative staining controls SLeX staining in cervical cancer tissue (**a**); SLeA staining in mamma carcinoma tissue (**b**), Lewis Y staining in physiological tissue of the Ileum (**c**)

Negative controls for each staining process are displayed in Fig. 1.

### Statistical analysis

IBM SPSS Statistics Version 26 was used for statistical investigations. To compare independent subgroups, we either used Mann–Whitney *U* test or Kruskal–Wallis test, whereas Spearman’s rank coefficient was used for correlation-analyses. In survival analyses for overall survival and progression-free survival, the Log-rank test was used to detect significant differences between Kaplan–Meier curves. Results with *p* < 0.05 were considered to be statistically significant.

## Results

### Sialyl Lewis X in endometrial cancer

**Fig. 2:**
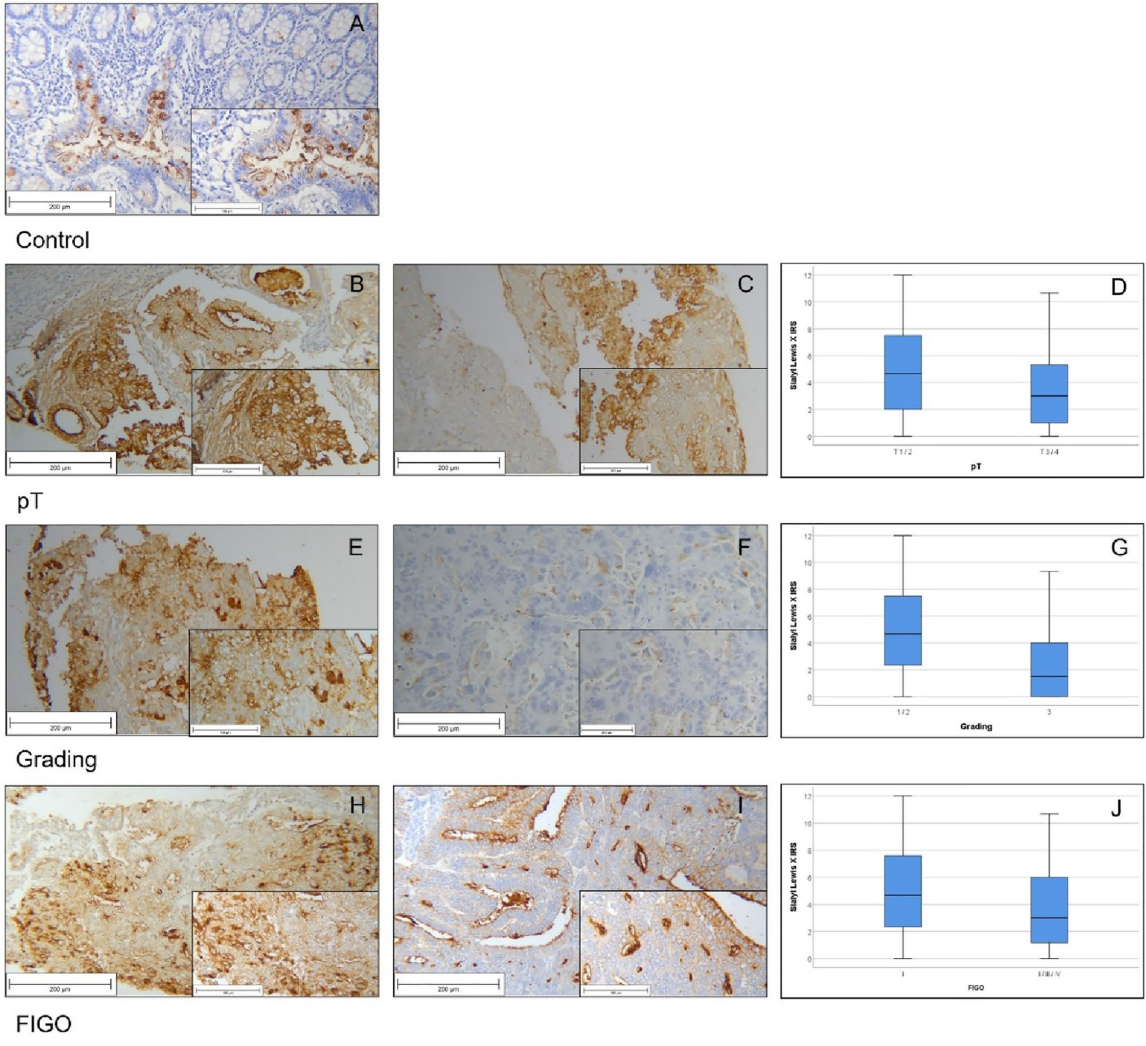
Results of SLeX staining. Physiological sigma tissue as positive staining control (**a**); cancer sample with smaller tumor size (pT1-2) with higher SLeX expression (**b**) and samples with bigger tumor size (pT3-4) and lower IRS (**c**) differed significantly from each other (*p* = 0.013; see boxplot; (**d**); low (G1) and moderate (G2) graded tissue showed higher SLeX intensity (**e**) than high-graded tumors (**f**). Boxplots of subgroups regarding grading (*p* < 0.001) (**g**); significant differences in SLeX expression were detected between FIGO I-subgroups (**h**) and FIGO II–IV (**i**) and are displayed in presented boxplot (*p* = 0.006; **J**)

Out of 234 patients, that were originally included in this study, we could only use 227 samples for scoring and statistical analysis of SLeX expression due to technical problems during the staining process. 4.5% of the included samples showed no or very low SLeX expression (IRS ≤ 1), whereas 13.7% showed an IRS of 9 or higher. The median IRS in our study group was 4.33. Thus 48% of the samples showed an IRS ≤ 4, whereas 52% scored above. To control the quality of our staining, we used physiological sigma tissue with moderate cytoplasmatic expression of SLeX as positive control (Fig. 2a).

When examining expression patterns of SLeX in the different subgroups, we made the following observations: the median IRS for specimens with smaller tumor sizes (pT1-2) was 4.66 and was significantly (*p* = 0.013) higher than the median IRS of 3.00 which we found in patients with higher T-status (pT3-4). Correlational calculations revealed a strong correlation of a high IRS with small tumor size (Spearman’s-Rho = − 0.166 with *p* = 0.013; Fig. 2b–d; Table 4). The same tendency could also be seen concerning the grade and FIGO-status. In high-grade cancer tissue (G3), we found a median IRS of 1.50, whereas cancer tissue of lower grade showed a median IRS of 4.67. This difference was also statistically significant (*p* < 0.001). In addition, IRS and grading also correlated significantly (Rho = − 0.268 with *p* < 0.001; Fig. 2e–g; Table 4). Examining FIGO-classification, specimens with FIGO I showed a median IRS of 4.67 and those with FIGO-status II and above showed a median IRS of 3.00. This difference was also tested to be statistically significant (*p* = 0.006) and presented with a strong Spearman correlation of Rho = − 0.183 with *p* = 0.006 between FIGO-status and SLeX expression (Fig. 2h–k; Table 4). Regarding N-Status and M-Status, we found no significant statistical differences or correlations in SLeX expression between groups with and without metastatic process.

**Table 4:** Overview of staining results and correlation analysis

	Sialyl Lewis X			Sialyl Lewis A			Lewis Y		
	Median IRS (± SD)	*p*	Spearman’s rho	Median IRS (± SD)	*p*	Spearman’s rho	Median IRS (± SD)	*p*	Spearman’s rho
*p*T									
T1-2	4.67 (± 3.19)	0.013*	− 0.166	4.00 (± 3.41)	0.013*	− 0.167	4.67 (± 2.79)	0.056	− 0.041
T3-4	3.00 (± 3.13)		(*p* = 0.013*)	1.50 (± 2.81)		(*p* = 0.013*)	4.00 (± 2.56)		(*p* = 0.553)
FIGO									
I	4.67 (± 3.19)	0.006*	− 0.183	4.00 (± 3.44)	0.345	− 0.064	4.67 (± 2.83)	0.527	− 0.043
II–IV	3.00 (± 3.13)		(*p* = 0.006*)	3.00 (± 3.14)		(*p* = 0.346)	4.42 (± 2.52)		(*p* = 0.528)
Grade									
I–II	4.67 (± 3.17)	< 0.001*	− 0.268	4.00 (± 3.38)	0.001*	− 0.218	5.00 (± 2.73)	0.005*	− 0.190
III	1.50 (± 2.81)		(*p* < 0.001*)	1.33 (± 2.58)		(*p* = 0.001*)	3.17 (± 2.59)		(*p* = 0.005*)
pN									
N0	4.50 (± 3.27)	0.652	− 0.049	4.00 (± 3.41)	0.553	− 0.067	4.00 (± 2.75)	0.038*	0.133
N1	4.00 (± 3.22)		(*p* = 0,458)	6.00 (± 3.81)		(*p* = 0.322)	6.25 (± 2.91)		(*p* = 0.051)
pM									
M0	4.33 (± 4.33)	0.413	0.016	3.00 (± 3.38)	0.964	0.004	4.00 (± 2.70)	0.520	0.126
M1	5.66 (± 3.38)		(*p* = 0.808)	4.00 (± 2.56)		(*p* = 0.948)	5.33 (± 2.73)		(*p* = 0.065)

Significant results are marked by *

**Fig. 3:**
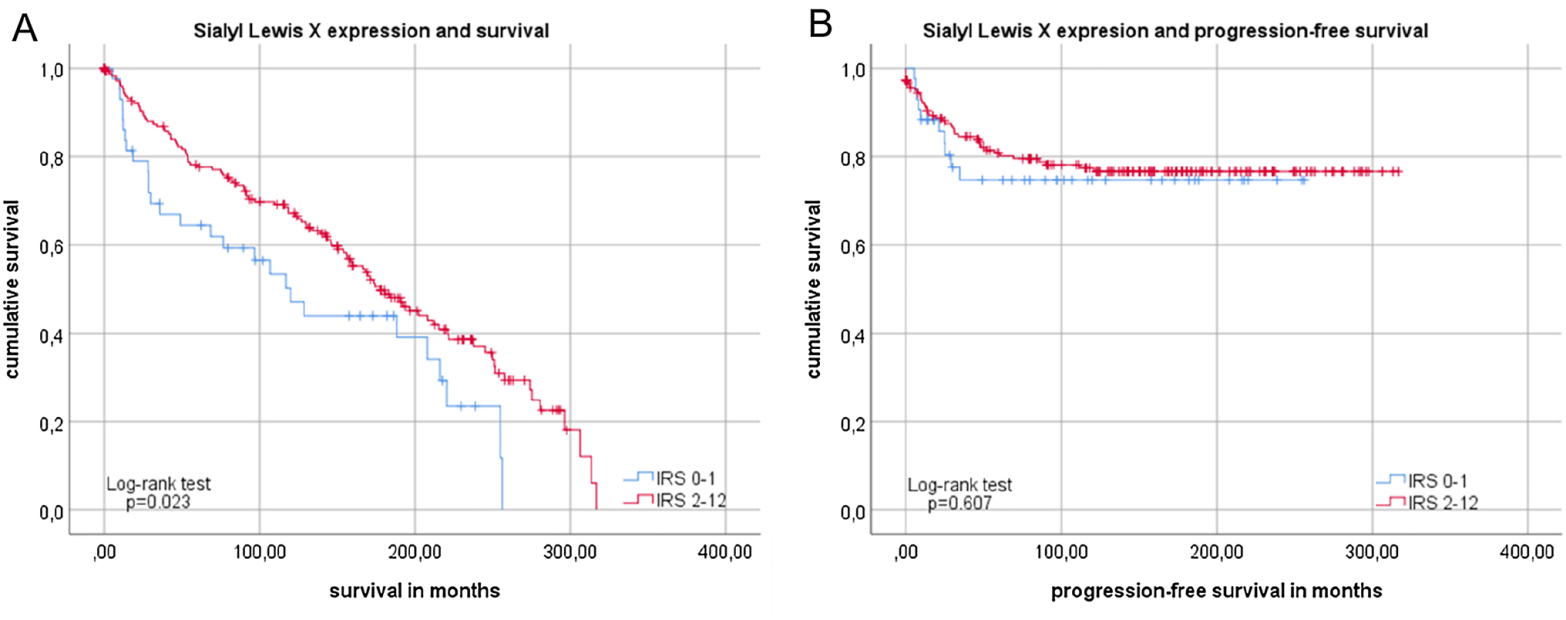
Survival analysis of SLeX expression Kaplan–Meier analyses for overall survival (**a**) and progression-free survival (**b**) with low SLeX expression (≤ 1; blue) and higher SLeX expression (≥ 2; red) in subgroup with all histological types. For distribution of patient groups, see Supplement 1

Survival analyses revealed an association of overall survival with SLeX expression. As displayed in Fig. 3, poor prognosis was associated with low IRS. Patients with specimens that showed very low SLeX expression IRS ≤ 1 died significantly (*p* = 0.023; Fig. 3a) earlier, than patients with higher SLeX expression (IRS ≥ 2). Although not statistically significant, similar tendencies could be seen in progression-free survival analyses. Patients with higher IRS (≥ 2) had a better outcome than patients with very low SLeX expression (*p* = 0.607; Fig. 3b).

### Sialyl Lewis A in endometrial cancer

**Fig. 4:**
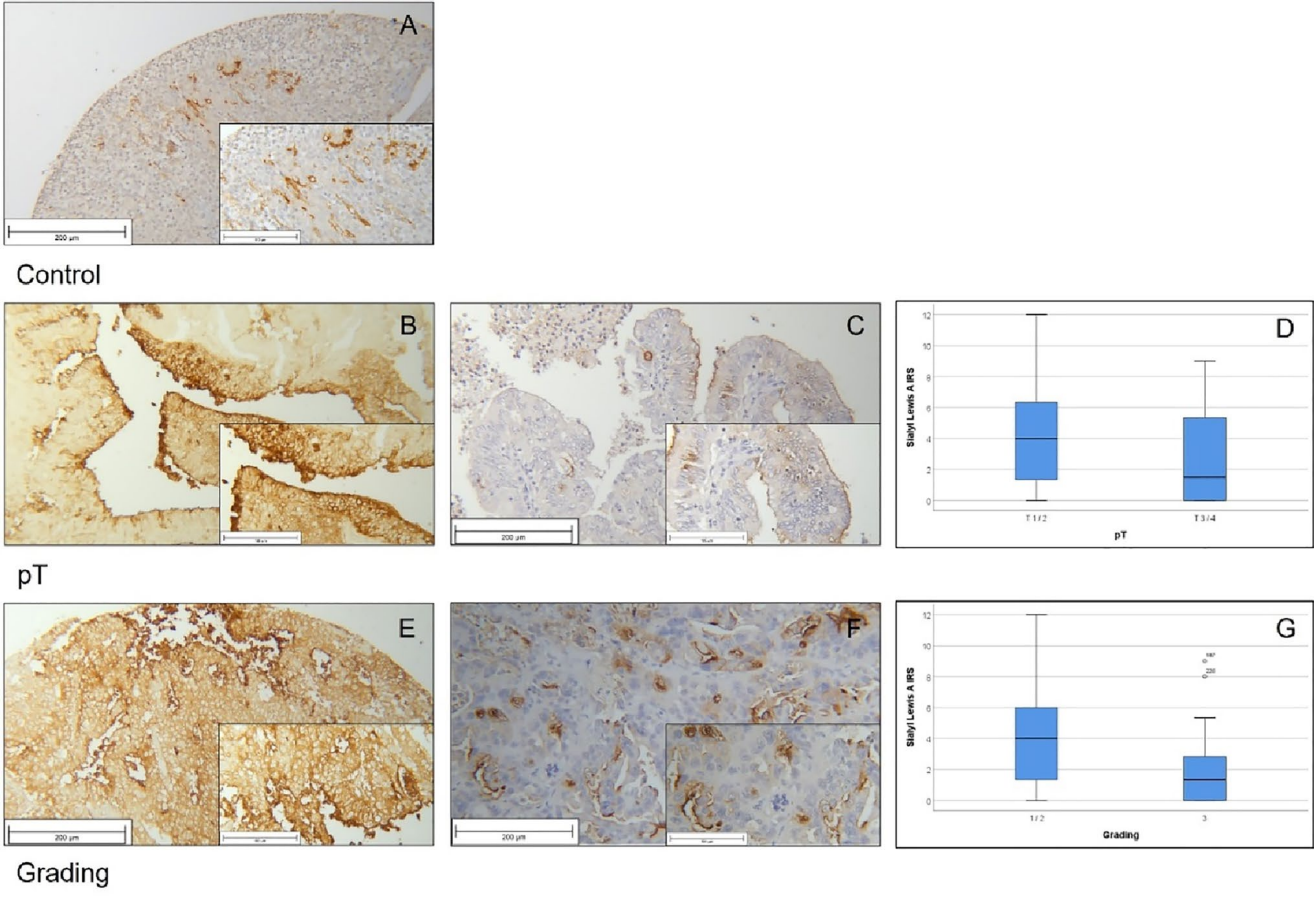
Results of SLeA staining SLeA staining of tonsil as positive control (**a**); smaller tumor size (T1-2) showed significantly higher SLeA expression (**b**) than smaller tumor sized tissue (**c**; *p* = 0.013). Both groups are depicted in boxplot (**d**); low and moderate graded (G1-2) tumors with high SLeA expression (**e**) and high-graded (G3) tumors (**f**) differed significantly (**g**; *p* = 0.001)

SLeA expression could be evaluated in 222 out of originally 234 included patients. Out of these samples a total of 36 (16.2%) showed no SLeA expression (IRS = 0), while an IRS of 9 and higher was scored in 30 (13.6%) cases. The median IRS of the cohort was 3.67. As quality control, we used tonsil tissue (Fig. 4a).

We found low SLeA expression (median IRS = 1.50) in specimens with bigger tumor size (pT ≥ 3). In comparison with the median IRS of 4.00, that was found in pT1-2, this difference was significant (*p* = 0.013) and showed a strong correlation Rho = − 0.167 (with *p* = 0.013; Table 4) between tumor size and SLeA expression (Fig. 4b–d).

Analyzing low and moderate grade tissue (G1-2), we found a median SLeA expression of 4.00. In contrast, the median IRS for cancer tissue from high-grade (G3) tumors turned out to be 1.33. This difference was significant with *p* = 0.001 (Table 4). A strong negative correlation between grading and SLeA expression was also detected (Spearman-Rho: − 0.218; *p* = 0.001; Table 4) (Fig. 4e–g).

By examining FIGO, pN-Status or pM-Status, no statistical differences in SLeA expression between the various subgroups were detectable. IRS values did not correlate significantly with any of the characteristics.

**Fig. 5:**
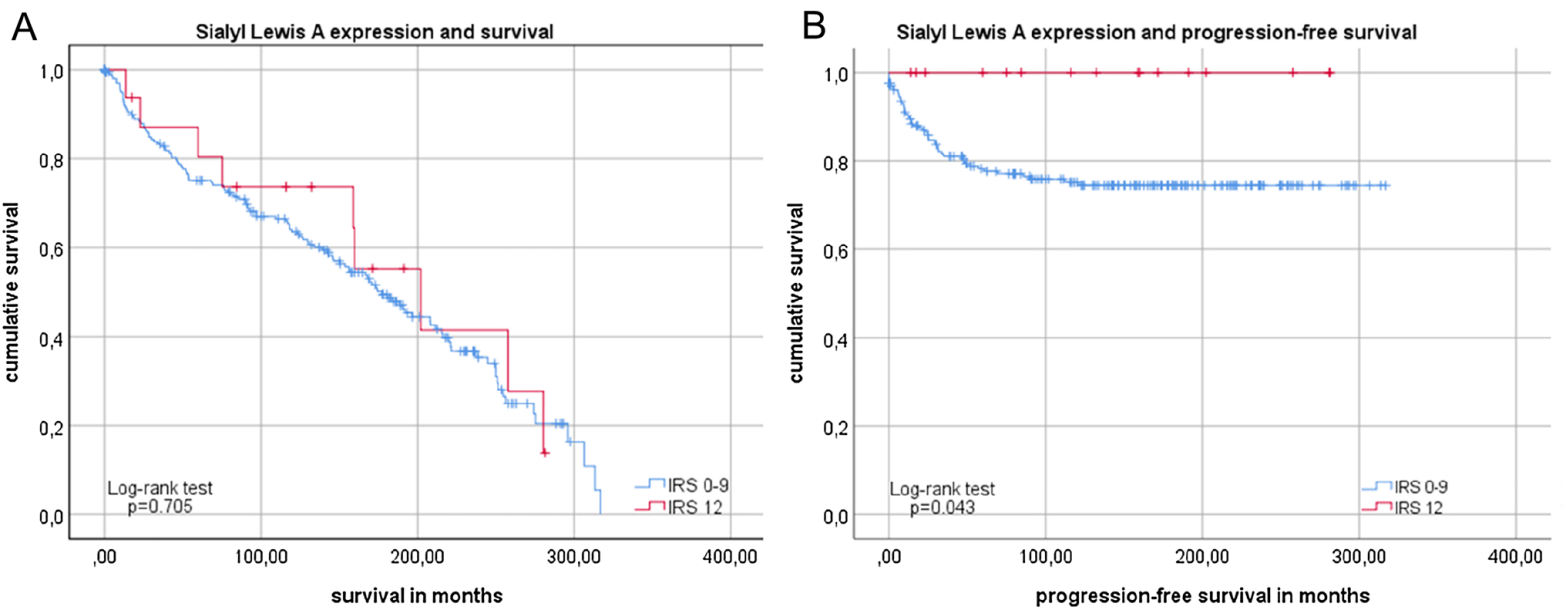
Survival analysis of SLeA expression Kaplan–Meier analysis of overall survival (**a**) without significant differences. Comparison of patients with high SLeA expression (IRS 12, red) and lower SLeA expression (IRS ≤ 9) in endometrial cancer regarding progression-free survival (**b**). For distribution of patient groups see Supplement 1

In analysis of overall survival, we could not detect a difference in survival time comparing after SLeA expression (*p* = 0.705; Fig. 5a).

Patients with high SLeA expression had better outcomes with regards to PFS: patients that had a high IRS (IRS = 12) presented with significantly (*p* = 0.043; Fig. 5b) fewer relapses—there was no event of progression in this group (Supplement 1).

### Lewis Y in endometrial cancer

**Fig. 6:**
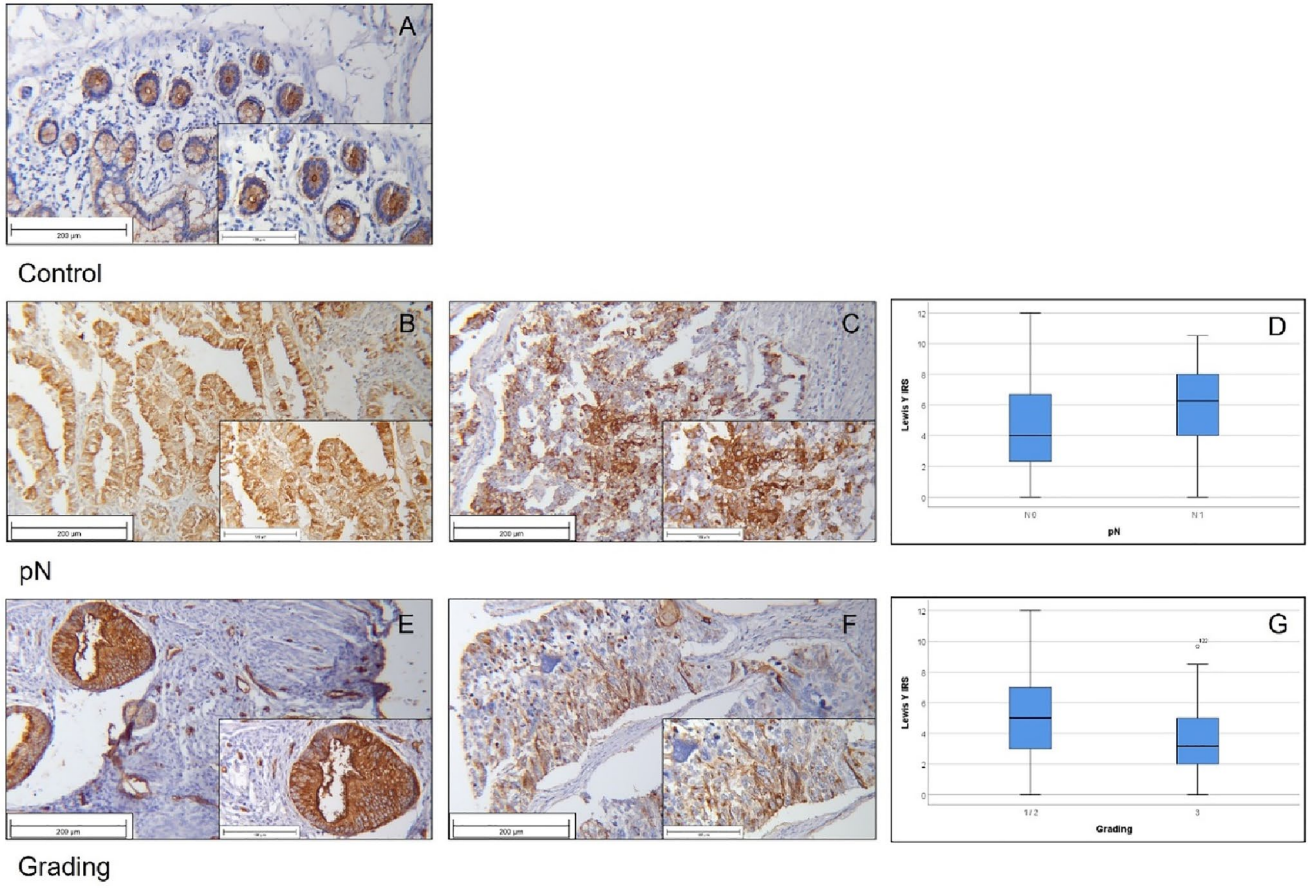
Results of LeY staining Normal, non-pathological Ileum tissue stained with Lewis Y as positive control (**a**); specimen without malignant lymph node involvement showed significant lower LeY expression (**b**) than samples with lymph node metastasis (**c**; *p* = 0.038). The difference is visualized with boxplot (**d**); specimen lower grading status (G1-2) presented high LeY expression (**e**), whereas high-graded tumors showed less LeY expression (**f**). This difference was also significant as shown in the boxplot (**g**; *p* = 0.005)

For the analysis of Lewis Y, 216 of 234 tissue samples could be used for further calculations. Ileum tissue was used for positive control (Fig. 6a).

Out of these 216 samples, 25 showed (11.6%) very low Lewis Y expression (IRS ≤ 1), whereas 7.5% only showed IRS 9 and higher. The all over median was 4.67.

Significant differences were detected within pN-Status and grade.

Patients with positive lymph nodes showed an enhanced Lewis Y expression (median IRS 6.25) and differed significantly (*p* = 0.038) from those without malignant lymph node involvement. Here, the median IRS was 4.00. A positive correlation between Lewis Y expression and lymph node phenotype could be detected with Rho = 0.133 but did not reach statistical significance (*p* = 0.051; Table 4; Fig. 6b–d).

Concerning grade contrary tendencies compared to the analyses above could be detected. In contrast to patients with low- and median-grade carcinomas (G1/2; median IRS 5.00), patients with high grade (G3) were tested to have a median IRS of 3.17. By testing with Mann–Whitney *U*, this difference was significant (*p* = 0.005; Table 4). High grading and low Lewis Y expression also correlated significantly (Rho = − 0.190 with *p* = 0.005; Table 4; Fig. 6e–g).

Analyzing IRS dissemination in subgroups of pM-, pT- and FIGO-status statistically significant variations in Lewis Y expression were not found.

**Fig. 7:**
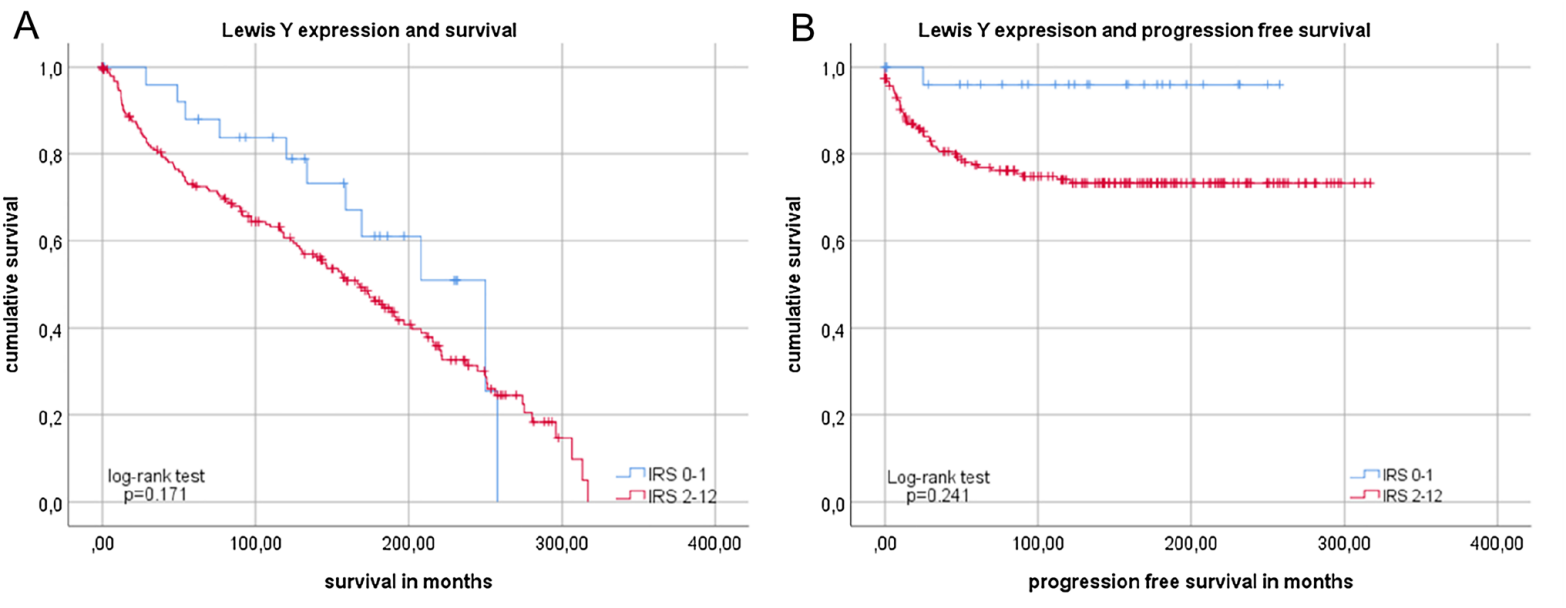
Kaplan–Meier analysis for overall and progression-free survival compared in regards of Lewis Y expression Subgroups with low expression (IRS ≤ 1, blue) and higher SLeA expression (IRS ≥ 2, red) differed significantly only in overall (**a**) and progression-free survival of patients (**b**). For distribution of patient groups, see Supplement 1

Whereas analyses showed a negative correlation between Lewis Y expression and grade patients with low Lewis Y expression (IRS ≤ 1) showed a tendency to better overall survival (Fig. 7a), but this was not significant (*p* = 0.171). A clear, significant association was detected for patients with low LeY expression regarding progression-free survival (*p* = 0.022; Fig. 7b). In the group of patients with low LeY expression, only one event (progression) took place during an observation of more than 20 years.

### Cox regression analysis

Cox regression analysis was used to test whether the parameters antigen expression (IRS of SLeX, SLeA and Lewis Y) and the clinicopathological variables were independent markers for overall and progression-free survival.

Regarding overall survival, age, grade, pT-and pN status turned out to be an independent factor (except pT in LeY-Cox-regression). The examined antigens were not independent (Table 5).

**Table 5:** Cox regression for overall survival (SLeX, SLeA, and LeY)

Sialyl Lewis X					Sialyl Lewis A					Lewis Y				
Var	*p*	HR	L95%	U95%	Var	*p*	HR	L95%	U95%	Var	*p*	HR	L95%	U95%
IRS 0–1 vs ≥ 2	0.722	1.089	0.681	1.742	IRS 0–9 vs ≥ 10	0.329	1.426	0.699	2.908	IRS 0–1 vs ≥ 2	0.055	1.877	0.986	3.573
Age	< 0.001*	1.062	1.039	1.085	Age	< 0.001*	1.068	1.045	1.092	Age	< 0.001*	1.061	1.038	1.084
Grade	< 0.001*	2.787	1.640	4.737	Grade	0.001*	2.502	1.475	4.244	Grade	< 0.001*	2.662	1.611	4.399
pT	0.011*	2.429	1.222	7.828	pT	0.040*	2.038	1.032	4.026	pT	0.053	1.991	0.990	4.001
pN	< 0.001*	1.530	1.243	1.883	pN	< 0.001*	1.498	1.213	1.849	pN	< 0.001*	1.508	1.219	1.865
FIGO	0.230	1.446	0.792	2.639	FIGO	0.181	1.509	0.826	2.754	FIGO	0.179	1.531	0.823	2.850

Regarding PFS, grade, pT, pN and FIGO-status seemed to be independent factors (except pT for Lewis Y Cox regression; Table 6).

**Table 6:** Cox regression for progression-free survival (SLeX, SLeA, and LeY)

Sialyl Lewis X					Sialyl Lewis A					Lewis Y				
Var	*p*	HR	L95%	U95%	Var	*p*	HR	L95%	U95%	Var	*p*	HR	L95%	U95%
IRS 0–1 vs ≥ 2	0.086	1.988	0.907	4.357	IRS 0–9 vs ≥ 10	0.972	0.000	0.000		IRS 0–1 vs ≥ 2	0.018*	11.27	1.503	84.528
Age	0.750	1.005	0.973	1.039	Age	0.606	1.003	0.971	1.037	Age	0.820	0.996	0.963	1.030
Grade	**0.001**	3.822	1.784	8.189	Grade	0.027*	2.365	1.103	5.071	Grade	0.001*	3.459	1.635	7.316
pT	**0.006**	3.392	1.431	8.044	pT	0.042*	2.455	1.033	5.833	pT	0.065	2.359	0.947	5.878
pN	**0.008**	1.615	1.131	2.306	pN	0.008*	1.639	1.137	2.362	pN	0.017*	1.556	1.083	2.236
FIGO	**0.028**	2.507	1.104	5.693	FIGO	0.043*	2.326	1.028	5.261	FIGO	0.018*	2.845	1.196	6.768

*Var* variables, *HR* hazard ratio, *L95%* lower 95% CI of exp(B), *U95%* upper 95% CI of exp(B)

Significant results are in bold and marked by *

## Discussion

In this study, we investigated the blood group antigens SLeX, SLeA and LeY in endometrial cancer, a disease with increasing incidence. A high expression of SLeX and SLeA correlated to better survival rates, while high expression of LeY went along with poorer prognosis. SLeX expression also correlated to low tumor stage: low pT-stage, low grade and low FIGO, resulting in better overall and progression-free survival. Interestingly, previous studies in other malignancies (for example lung (Iwanari et al. 1990) and breast cancer (Jeschke et al. 2005)), suggested that high levels of SLeX are associated with advanced disease, so researchers suggested it to be a tumor marker. High levels of SLeX were associated with worse prognosis in liver cancer (Nakagoe et al. 2002).

SLeX can promote metastasis by inducing overexpression of E-selectin, resulting in haematogenous metastasis (Okuno et al. 2003; Shah et al. 2009; Jin and Wang 2020). Also in cervical carcinoma in situ, the expression of SLeX is higher compared to healthy cervical tissue (Engelstaedter et al. 2012). In contrast, in invasive carcinoma, a loss of SLeX was reported (Moro-Rodríguez and Álvarez-Fernández 2008), leading to the opposite effect of SLeX: it can also improve the anti-tumor immune response; its derivate 6-sulfo SLeX is involved in the recruitment of T-lymphocytes, which was shown in urothelial carcinomas (Taga et al. 2015). High SLeX levels can also support natural killer cells attacking tumor cells (Ohyama et al. 2002). This might result in lower tumor stages and better survival rates, matching our results. Based on the paradoxical observations, Ohyama et al. suggest that the results of SLeX expression depend on its different expression levels (Ohyama et al. 2002). Jin et al. found out that in early tumor stages, the abnormal SLeX synthesis leads to an immune imbalance, while in advanced stages, it promotes tumor vascularization and metastasis (Jin and Wang 2020). In summary, the exact role of SLeX and the underlying mechanisms are not clearly understood yet. Nevertheless, it seems clear that abnormal glycolization plays an important role and data supporting a suppressing and a promoting role in tumorigenesis exist makes more investigations necessary.

Like SLeX, also its isomer SLeA serves as a tumor marker: as epitope of the Ca 19-9 antigen, it is overexpressed in pancreatic, colorectal and breast cancer (Ugorski and Laskowska 2002; Trinchera et al. 2017; Jeschke et al. 2005). Analogous to SLeX, also SLeA is a ligand for E-selectin and an overexpression of both—E-selectin and SLeA or SLeA alone is associated with distant haematogenous metastasis (Kannagi 2004, 2007; Tozawa et al. 2005). In contrast to these described results, we detected a high expression of SLeA being associated with low pT-stage, low grade and better overall survival rates. Until now, only little data about SLeA in EC exist: Inoue et al. described an increased expression of SLeA in EC compared to healthy endometrial tissue (Inoue et al. 1987). As with SLeX, the cellular mechanisms of SLeA expression are not completely understood. In cervical cancer for example, it was shown that the effect of SLeA depends on the tumor stage: in CIN II, a high expression was correlated to a progressive state (Kolben et al. 2017), while in invasive carcinomas, a loss of SLeA was shown (Moro-Rodríguez and Álvarez-Fernández 2008), resulting in a poorer prognosis with low SLeA expression.

Similar to SLeX and SLeA, also LeY is overexpressed in several tumors, such as breast cancer, ovarian cancer, and prostate and colon cancers (Arai and Nishida 2003; Madjd et al. 2005). At the same time, the expression of LeY has been described to be associated with poorer prognosis (Madjd et al. 2005; Liu et al. 2018). In EC, high expression of LeY went along with positive lymph node status—a predictor for poorer prognosis. It also correlated significantly to poorer progression-free survival, but not to overall survival in EC. Different theories about the underlying mechanism exist: Liu et al. suggest a connection to chemo-resistance, as in chemo-resistant ovarian cancer cells, LeY was significantly elevated (Liu et al. 2018). In addition, ovarian cancer cells with high LeY expression developed more often chemo-resistance (Liu et al. 2018). Both observations resulted in progression and highlight the role of LeY in invasion and metastasis of ovarian cancer (Liu et al. 2019, 2009; Iwamori et al. 2005; Yan et al. 2010). In addition, the proliferation of cancer, e.g., ovarian cancer, can be influenced by LeY through the PIK3/Akt pathway (Liu et al. 2009). In addition, in colon cancer, LeY is examined: its expression led to a decrease of apoptosis (Baldus et al. 2006), also resulting in progression and thus poorer survival rates. In cervical cancer and its pre-cancer lesions, LeY has a prognostic impact: significant differences were found between normal samples compared to CIN I/CIN II/CIN III and invasive cancer (Engelstaedter et al. 2012).

Our results show significant correlations of SLeX, SLeA and LeY to clinicopathological variables and survival rates independent of molecular subtypes. Lewis Y turned out to be independent variables regarding progression-free survival, and SLeX and SLeA showed a significance regarding survival rates, but they were not independent factors. Beside Lewis Y, also SLeA turned out to be a possible marker for progression-free survival: in the group with better progression-free survival rates, we had almost no events. As we used immunohistochemical methods, we cannot say if these results are consequence or cause of the cancer phenotype. Further experiments are, therefore, needed and could lead to a deeper understanding of the molecular mechanisms. Finally, this could result in the identification of new therapeutic targets for endometrial cancer treatment.

## Conclusions

This is the first study examining the expression of SLeX, SLeA and LeY in endometrial cancer. The examined structure could be used as potential prognostic marker in EC in the future. This is a descriptive analysis only, so further studies exploring the underlying mechanisms on cellular level are warranted.

## Supplementary Information

Below is the link to the electronic supplementary material.

Supplementary file1 (DOCX 16 KB)

## Data Availability

The datasets generated during and analyzed during the current study are not publicly available but are available from the corresponding author on reasonable request.

## Abbreviations

CIN: Cervical intraepithelial neoplasia
EC: Endometrial cancer
LeY: Lewis Y
OS: Overall survival
PFS: Progression-free survival
SLeA: Sialyl Lewis A
SLeX: Sialyl Lewis X
