# Supplementary material for: Blood group antigens SLeX, SLeA, and LeY as prognostic markers in endometrial cancer
Source: J Cancer Res Clin Oncol. 2022 Jun 21;148(12):3323–35. doi: 10.1007/s00432-022-04098-8 (PMC9587092; doi:10.1007/s00432-022-04098-8)
Supplement: Supplementary file 1 — Supplementary file1 (DOCX 16 KB) [file 432_2022_4098_MOESM1_ESM.docx]

Blood group antigens SLeX, SLeA, LeY as prognostic markers in endometrial cancer

Journal: Journal of Cancer Research and Clinical Oncology

Thomas Kolben^1#^, Lena Müller^1#^, Sarah Meister^1^, Lucia Keilmann^1^, Christina Buschmann^1^, Fabian Trillsch^1^, Alexander Burges^1^, Bastian Czogalla^1^, Sophie Mitter^1^, Elisa Schmoeckel^2^, Stefanie Corradini^3^, Sven Mahner^1^, Udo Jeschke^1,4,^ *, Mirjana Kessler^1^, Susanne Beyer^1^

***** Correspondence: Udo.Jeschke@med.uni-muenchen.de; Tel.: +49-89-44007-4531

**Supplement 1: Size of patient groups regarding the IRS of SLeX, SLeA, LeY**

|  |  | **Total number** | **Number of events** | **Censored** |
| --- | --- | --- | --- | --- |
| **SLeX - OS** | IRS ≤1 | 43 | 28 | 15 |
|  | IRS >1 | 184 | 104 | 80 |
| **SLeX - PFS** | IRS ≤1 | 43 | 10 | 33 |
|  | IRS >1 | 184 | 39 | 145 |
| **SLeA - OS** | IRS ≤9 | 206 | 120 | 86 |
|  | IRS >9 | 16 | 9 | 7 |
| **SLeA - PFS** | IRS ≤9 | 206 | 48 | 158 |
|  | IRS >9 | 16 | 0 | 16 |
| **LeY - OS** | IRS ≤1 | 28 | 11 | 16 |
|  | IRS >1 | 189 | 118 | 71 |
| **LeY - PFS** | IRS ≤1 | 27 | 1 | 26 |
|  | IRS >1 | 189 | 46 | 143 |
